# A veterinary survey of factors associated with capture-related mortalities in cheetahs (*Acinonyx jubatus*)

**DOI:** 10.4102/jsava.v90i0.1723

**Published:** 2019-07-31

**Authors:** Cindy Braud, Emily P. Mitchell, Vincent van der Merwe, Adrian S.W. Tordiffe

**Affiliations:** 1 Centre de Coopération Internationale en Recherche Agronomique pour le Développement (CIRAD), UMR ASTRE, Campus International de Baillarguet, Montpellier, France; 2 National Zoological Gardens of South Africa, Pretoria, South Africa; 3 Endangered Wildlife Trust, Carnivore Conservation, Modderfontein, South Africa; 4 Department of Paraclinical Sciences, Faculty of Veterinary Science, University of Pretoria, Onderstepoort, South Africa

**Keywords:** *Acinonyx jubatus*, cheetahs, capture, immobilisation, survey, mortalities

## Abstract

The objective of this study was to gain better insight into factors associated with the capture-related mortality rate in cheetahs. A link to an online questionnaire was sent to zoo and wildlife veterinarians through the Species Survival Plan Programme and European Endangered Species Programme coordinators and via the ‘Wildlife VetNet’ Google group forum. The questionnaire consisted of 50 questions relating to the veterinarians’ country of residence and experience, the medicine combinations used, standard monitoring procedures, capture-related complications and mortalities experienced in this species under different capture conditions. In addition, necropsy data from the national wildlife disease database of the National Zoological Gardens of South Africa were examined for cases where anaesthetic death was listed as the cause of death in cheetahs. A total of 75 veterinarians completed the survey, with 38 from African countries and a combined total of 37 from Europe, the United States (US) and Asia. Of these, 24% (*n* = 18/75) had experienced at least one capture-associated cheetah mortality, with almost all of the fatalities (29/30) reported by veterinarians working in Africa. A lack of anaesthetic monitoring and the absence of supplemental oxygen were shown to be significant risk factors for mortality. Hyperthermia, likely to be associated with capture stress, was the most common reported complication (35%). The results suggest that free-ranging rather than habituated captive cheetahs are particularly at risk of dying during immobilisation and transport. The capture-related fatalities in this species do not appear to be associated with either the veterinarian’s level of clinical experience or the immobilisation agents used.

## Introduction

In the last few decades, cheetahs (*Acinonyx jubatus*) have suffered drastic population declines because of several anthropogenic factors that now confine them to a mere 9% of their historical distribution range. More than half of the world’s estimated 7100 free-ranging individuals currently reside in six southern African countries (Durant et al. 2016).

In South Africa, the majority of the estimated 1200 free-ranging cheetahs are confined to national and provincial parks and a narrow stretch of farmland along the country’s northern border (Van der Merwe et al. 2016). A smaller but growing number of cheetahs have been re-introduced to smaller private or state-owned reserves scattered across the country. Since 2011, these animals have been managed through the Endangered Wildlife Trust’s Cheetah Metapopulation Project, increasing their numbers from 240 cheetahs on 40 reserves to 330 animals on 54 reserves. The project primarily aims to maintain the genetic diversity of the cheetahs in these small reserves and this requires the occasional immobilisation of individuals for various management and translocation purposes.

Since its inception, however, 23 capture-related deaths have been reported, amounting to 20% of the total of 114 cheetahs immobilised. These capture procedures were carried out by different veterinarians with varying levels of general wildlife clinical experience. Unfortunately, only a few detailed incident and post-mortem reports were submitted for these cases and it was thus not possible to draw any clear conclusions regarding the underlying causes of the fatalities. This high mortality rate seriously threatens the future viability of the project.

Although some mortalities are inevitable during chemical capture and anaesthesia of wildlife species, rates exceeding 2% should be considered unacceptable in any large mammalian species (Arnemo et al. 2006). A capture-related mortality rate in cheetahs that exceeds this limit by 10-fold is therefore of critical concern, especially in a species of such conservation importance. To better understand the potential reasons for these mortalities, we conducted an online anonymised survey amongst zoo and wildlife veterinarians who have immobilised both captive and free-ranging cheetahs in their careers. We also reviewed all the available post-mortem reports of cheetahs that died unexpectedly during or soon after immobilisation. The results of the survey and post-mortem findings are reported in this study.

## Materials and methods

A link to an online questionnaire was circulated to wildlife and zoo veterinarians through the Species Survival Plan Programmes and European Endangered Species Programme coordinators and via the ‘Wildlife VetNet’ Google group forum. A covering letter, included with the survey, explained the objectives of the study and how to correctly answer the questions. The letter also assured the participants that their answers would remain anonymous.

The questionnaire consisted of 50 questions that required either a checkmark, numeric answers or comments. Questions 1–11 asked about the veterinarians’ year of graduation, country of residence and experience in cheetah immobilisation. Questions 12–23 asked about common chemical capture protocols and anaesthetic monitoring used in different situations. Questions 24–50 asked about the objective of the immobilisation, the complications and the mortalities experienced with this species. Microsoft Excel was used to create a database and to enter the data from the survey. In addition, as few necropsy reports were available for the cheetahs’ mortalities reported in response to the questionnaire, the wildlife disease database of the National Zoological Gardens of South Africa (NZG) was examined for cases where anaesthetic death was listed as the cause of death in cheetahs (*n* = 13) and the lesions listed in the necropsy reports were recorded.

Univariate analyses were conducted for each potential predictor using a general linear model. As it was impossible to gather anaesthetic records for each capture event, stepwise regressions were conducted to determine the best-fit model for predicting outcomes. The multivariate best-fit model considered all variables that were significant predictors of the outcome in the univariate analyses. Odds ratios (ORs) and 95% confidence intervals (CIs) were calculated for each potential risk factor. Odds ratios measured the association between a risk factor and peri-anaesthetic death. Risk factors were those with OR > 1 and protective factors had OR < 1. All analyses were performed with R Studio (Boston, MA, United States). A *p*-value of less than 0.05 was considered significant.

### Ethical considerations

The survey study was approved by the National Zoological Gardens of South Africa’s Research and Ethics Committee (P17/06) and by the Animal Ethics Committee of the University of Pretoria (V025-17).

## Results

Completed online surveys were received from 75 veterinarians: 50.7% (38/75) were from Africa, 36.0% (27/75) from Europe and Asia and 13.3% (10/75) from North America. More than half (21/38; 55.3%) of the veterinarians from Africa who completed the survey had been practising for more than 10 years, while just fewer than a third (12/37; 32.4%) of veterinarians from Europe, North America and Asia had been practising for this length of time. Table 1 provides a summary of the occupational experience of veterinarians with regard to the number of years since graduation and the number of cheetahs they had immobilised during their careers, as well as the total number of cheetah mortalities reported by each group.

**TABLE 1:** Numbers (and percentages in each category) of surveyed veterinarians practising in African countries (*n* = 38) and combined numbers in Europe, North America and Asia (*n* = 37) in each time period since qualifying as a veterinarian (above) and in categories that reflect the number of cheetahs immobilised during their careers (below), as well as the total number of deaths reported in each group.

Variable	Africa			EU, US and Asia		
	Veterinarians		No. of deaths	Veterinarians		No. of deaths
	*n*	%		*n*	%	
Years qualified						
< 5 years	12	31.6	0	10	27.0	0
5–10 years	5	13.2	1	15	40.5	3
> 10 years	21	55.3	32	12	32.4	0
Cheetahs immobilised						
< 10 cheetahs	8	21.1	0	16	43.2	0
10–50 cheetahs	10	26.3	6	15	40.5	0
> 50 cheetahs	20	52.6	27	6	16.5	3

EU, Europe; US, United States.

Just about half (36/74; 48.6%) of the responses were from veterinarians working in zoos with the majority of these practising in Europe (26/36; 72.2%) and North America (10/36; 27.8%). Twenty-seven private wildlife practitioners completed the survey (27/75; 36.5%), with the majority (26/27; 96.3%) working in Africa.

**FIGURE 1:**
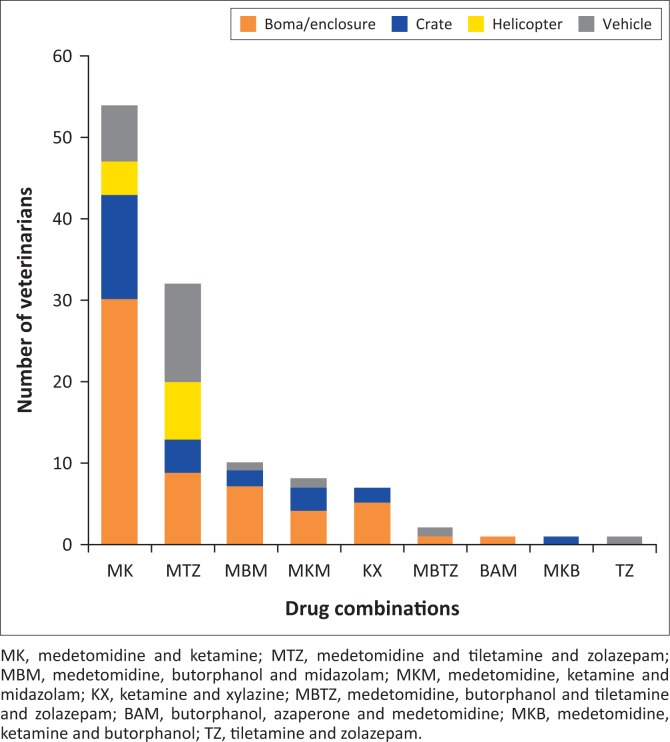
Stacked bars showing the number of veterinarians that reported the use of different combinations of medicines used to immobilise cheetahs under various capture conditions.

Ketamine and medetomidine (2.37–3.25/0.048–0.073 mgkg^−1^) was the combination most often used for chemical capture (47.0%) under various captive and free-ranging conditions, followed by medetomidine and a tiletamine-zolazepam combination (0.04–0.06/1.20–1.54 mgkg^−1^) (27.9%) and medetomidine, butorphanol and midazolam (0.031–0.039/0.20–0.26/0.13–0.18 mgkg^−1^) (8.7%) (Figure 1).

Supplemental anaesthesia was often achieved with intravenous (IV) ketamine (0.84-1.43 mgkg^−1^) in 63.5% or medetomidine (0.014-0.02 mgkg^−1^) in 19.1% of cases. Maintenance of anaesthesia, through isoflurane inhalation (1.0%–4.0%) was provided in 28.4% of cases.

Almost a quarter (21.7%) of the veterinarians did not record any of the basic physiological parameters [heart rate, respiratory rate, rectal temperature, blood oxygen saturation (SpO_2_) or end-tidal carbon dioxide (EtCO_2_)] during chemical capture. When monitoring was actively performed, respiratory rate (97.8%), rectal temperature (95.6%) and heart rate (94.5%) were the parameters they most often recorded. Oxygen saturation (61.5%) and an assessment of dehydration status (40.66%) were partially recorded, whereas capnography and electrocardiography (ECG) data were almost never recorded. Oxygen supplementation and fluid administration (subcutaneous or IV route) were only provided in 27.8% and 47.0% of cases, respectively.

Twenty-four per cent (*n* = 18/75) of veterinarians had faced at least one capture-associated cheetah mortality in their career. The majority of the deaths were experienced by veterinarians working in Africa (*n* = 17/18; 94.4%). Of these, 14/18 (77.8%) had immobilised more than 50 cheetahs in their careers. Most encountered only a single death (*n* = 12/18), one faced two deaths, three experienced three deaths and one faced a total of nine fatalities.

**FIGURE 2:**
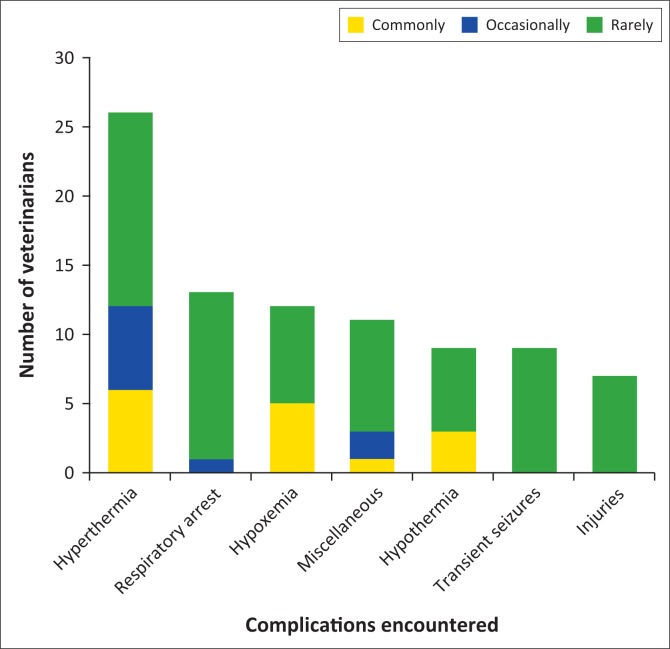
Stacked bars showing the number of veterinarians who had experienced various complications, and the perceived frequency of such events, during the capture and anaesthesia of cheetahs in their careers. Commonly = perceived to occur in > 50% of captures; Occasionally = perceived to occur in between 25% and 50% of captures; Rarely = perceived to occur in < 25% of captures.

Amongst the veterinarians, 43.0% reported that they had not experienced chemical capture-associated complications in cheetahs. Hyperthermia was the most common complication reported (35.0%), followed by respiratory arrest (17.3%), hypoxaemia (16.0%), hypothermia (12.0%), transient seizures (12.0%), trauma (9.3%) (Figure 2). The miscellaneous complications reported included vomition, aspiration, prolonged recovery, tachycardia, bradycardia and collar-related problems.

## Reported mortalities

We received data through the survey for 30 capture-related cheetah mortalities from 18 veterinarians. The records included three juveniles (< 1 year), five subadults (< 2 years) and 22 adults, corresponding to 23 male cheetahs and 7 female cheetahs. The majority of deaths were reported in individuals captured for translocation purposes (17/30) followed by cheetahs immobilised for health examinations (6/30), other procedures (4/30) and the fitting of telemetric collars (3/30). The majority (43.3%; *n* = 13/30) were immobilised in an enclosure or boma or from a vehicle (26.67%; *n* = 8/30), while relatively few were immobilised in a transport crate (20%; *n* = 6/30) or from a helicopter (10%; *n* = 3/30). A large proportion of the cheetahs that died were subjectively judged by the surveyed veterinarians to be stressed at the time of capture (20/30; 66.7%), whereas 16.67% (5/30) were considered calm and 16.67% (5/30) alert.

No specific medicine, dose or any of the medicine combinations were statistically associated with the mortalities. Medetomidine and tiletamine-zolazepam (0.051 ± 0.017/1.090 ± 0.260 mgkg^−1^) was the combination administered most in cheetahs that died (*n* = 10/30). This is followed by the use of tiletamine-zolazepam alone (3.954 ± 0.768 mgkg^−1^) (*n* = 8/30), ketamine and xylazine (2.736 ± 0.77/3.42 ± 0.962 mgkg^−1^) (*n* = 6/30), medetomidine and ketamine (0.067 ± 0.025/4.356 ± 3.517 mgkg^−1^) (*n* = 5/30) and medetomidine, butorphanol and midazolam (0.034/0.231/0.143 mgkg^−1^) (*n* = 1/30). Only one of the cheetahs received a specific tranquilliser prior to transportation (10 mg of diazepam).

No anaesthetic monitoring was performed for 5 of the 30 cheetahs. Respiratory rate and rectal temperature were the only values recorded for 17 and 21 of the animals, respectively. Where rectal temperature had been recorded, hyperthermia (> 40 °C) was noted in 61.9% of cases (*n* = 13/21). Six individuals had rectal temperatures between 39 °C and 40 °C (28.57%) and only two were lower than 39 °C (9.52%). Extreme hyperthermia with rectal temperatures of 43 °C or above was noted in three cases. For cheetahs that presented with hyperthermia, cooling was attempted by pouring water on the animal in 12/13 cases. Intravenous fluid therapy was provided to 43.3% (13/30) of animals that died and 16.67% received oxygen supplementation. The majority of deaths 12/30 (40%) occurred at least 1 hour after immobilisation, 9/30 (30%) after antidote administration, 7/30 (23.3%) between 30 minutes and 1 h after immobilisation and only 2/30 (7%) within the first 30 min of immobilisation.

Necropsy reports of 13 cheetahs (seven female cheetahs, six male cheetahs) were collected from the NZG wildlife disease database. In those cases, a complete set of tissues was not submitted for examination and autolysis of some individuals precluded data evaluation in detail. Body condition, where noted, was described as good (*n* = 2) or excellent (*n* = 3). In 10 cases, variable combinations of tissue congestion, pulmonary oedema, renal tubular necrosis, periacinar hepatocellular degeneration or necrosis were present. Terminal aspiration of stomach contents was seen in six cases and tracheal haemorrhage, alveolar atelectasis or haemorrhage in three cases. Suspected bronchospasm was seen in two cases and laryngeal oedema in one case. There was no evidence of pre-existing significant pathology in seven cases. Five animals had pre-existing disease that may have played a role in their deaths including four animals with septicaemia and/or toxaemia, one of which also had hepatic veno-occlusive disease and mild chronic renal disease, while another also had mild myocardial fibrosis. One other cheetah had mild myocardial fibrosis. One cheetah had widespread vasculitis of unknown cause and died of cardiac tamponade. Enlarged and/or hyperplastic adrenal glands were noted in six animals. Subacute lymphoplasmacytic gastritis and mild chronic renal disease were each noted in five animals and gastric ulcers and splenic myelolipomas each in one animal.

The multivariate logistic regression model created to examine the relationship between veterinarians’ characteristics, immobilisation medicine combinations and capture complications identified two risk factors associated with mortality. A failure to record and monitor physiological parameters during chemical capture (OR, 4.66; 95% CI, 1.33–22.2; *p* = 0.027) and partial monitoring, without the evaluation of SpO_2_ (OR, 2.41; 95% CI, 1.10–5.43; *p* = 0.03), was significantly associated with increased risk. Oxygen supplementation was shown to be a protective factor (OR, 0.12; 95% CI, 0.04–0.34; *p* = 0.0014). Combinations of medicines, veterinarian experience and chemical capture conditions (boma vs. helicopter vs. vehicle vs. crate) were not considered significant risk factors for mortality.

## Discussion

Observations from the present survey demonstrate that the vast majority of veterinarians who experienced capture-related mortalities in cheetahs practised in African countries where cheetahs occur both in captive and free-ranging environments. This may, to some extent, be a reflection of the larger number of cheetahs immobilised in this continent, but as we did not ask for information on the actual number of cheetahs immobilised per year, we were not able to assess the relative incidence of mortalities in each region. Veterinarians from African countries have approximately equal experience in the chemical capture of captive versus free-ranging cheetahs, whereas veterinarians from non-African countries largely work with cheetahs in zoo environments. Captive cheetah facilities in African countries differ quite substantially in terms of their enclosure design and animal management from typical zoos. Cheetah enclosures in Africa are often large and the levels of human and animal interactions at these facilities vary tremendously. The access to veterinary anaesthesia and monitoring equipment is also likely to be limited compared to what is available in zoo veterinary clinics and hospitals. The veterinarians from Africa were, however, not inexperienced. More than half of those who responded to the survey had practised for more than 10 years and had captured more than 50 cheetahs in their careers. This group of veterinarians nevertheless experienced the majority of the mortalities. Overall, the results do indicate that unlike the situation in African countries, capture-related mortalities are not a significant problem in captive zoo cheetahs in Europe and North America. Furthermore, all the cheetahs that died in the Endangered Wildlife Trust’s Cheetah Metapopulation Project were considered to be free-ranging. These combined results thus suggest that free-ranging cheetahs are particularly at risk of capture-related complications.

Amongst all veterinarians, hyperthermia was considered the primary complication observed during anaesthesia. Although the linear model did not indicate hyperthermia as a significant risk factor for mortality during capture, it may nevertheless have played a role, because rectal temperatures were not recorded in all cases. Capture-associated hyperthermia is a common sequel during the capture of wild ungulates and has been shown to largely be a stress response rather than being associated with high environmental temperatures or physical exertion (Meyer et al. 2008). In one study in Scandinavia, where environmental temperatures are relatively low, hyperthermia was thought to have contributed to capture-related mortalities in 9 out of 10 brown bears (*Ursus arctos*), three out of nine Eurasian lynx (*Lynx lynx*) and two out of three grey wolves (*Canis lupus*) (Arnemo et al. 2006). In some captive facilities, cheetahs have been shown to suffer from chronic stress (Terio, Marker & Munson 2004) and are often described as ‘highly strung’. The effects of acute stress during capture, however, have, to our knowledge, not been studied in cheetahs. The link between stress and hyperthermia in cheetahs was demonstrated to some extent in a study in which free-ranging cheetahs were shown to develop an acute, but temporary spike in body temperature immediately after successful hunts. This acute elevation in body temperature also occurred in coalition cheetahs that did not participate in the hunt and was far less pronounced when hunts were unsuccessful. This suggests that the hyperthermia experienced is largely independent of physical exertion. The authors attributed the hyperthermia to the stress associated with the high level of vigilance that cheetah seem to display at kills – perhaps in fear of attracting other more dominant predators such as spotted hyenas (*Crocuta crocuta*), leopards (*Panthera pardus*) and lions (*Panthera leo*) (Hetem et al. 2013). The subjective assessment in our survey of high levels of stress in 20 of the 30 cheetahs that died also suggests that acute psychological stress may be an important factor that could potentially contribute towards capture mortalities. In addition, seven (54%) of the cheetahs for which necropsy data were available had evidence of chronic stress (lymphoplasmacytic gastritis, gastric ulcers, myocardial fibrosis, chronic renal disease, splenic myelolipomas and adrenocortical gland hyperplasia) (Citino & Munson 2005; Gillis-Germitsch et al. 2017; Munson 1993; Terio, Munson & Moore 2012), which may have primed them for stress-related responses to anaesthesia.

It is therefore of critical importance that cheetah captures and translocations are carefully planned to minimise any potential stressors to the animals. If such stressors cannot be avoided, then the veterinarian should either recommend against continuing with the procedure or should have appropriate medicines and equipment available to deal with potential complications, including severe hyperthermia. Sawicka et al. (2015) found that in immobilised blesbok (*Damaliscus pygargus phillipsi*), the use of icepacks and dousing with copious amounts of water (between 4 °C and 28 ᵒC) were the most effective methods of reducing body temperature. The peripheral vasoconstrictive and impaired thermoregulatory effects of the α-2 agonists may, however, significantly impede active cooling techniques. Tordiffe (2017) reported that aggressive cooling in cheetahs with capture-related hyperthermia (> 40 ᵒC) with cold water dousing and the use of a leaf blower stabilised them, but failed to significantly reduce their rectal temperatures until they were intubated and given isoflurane in oxygen. Isoflurane is known to be a potent vasodilator, which may facilitate heat loss during active cooling, but its use requires anaesthetic equipment that may be difficult to provide in the field. The use of α-2 antagonists may also aid in heat loss through peripheral vasodilation and the return of thermoregulatory functions. However, this will also result in arousal, which may, in turn, cause significant ongoing stress to the animal if it is not allowed to recover in a quiet undisturbed environment. Hyperthermia is also known to increase metabolic rate and oxygen demand. In rats, oxygen demand increased by 5% – 6% for every 1 ᵒC increase in body temperature (Carlsson, Hägerdal & Siesjö 1976). Cheetahs with hyperthermia are therefore likely to benefit from the provision of supplementary oxygen.

Interestingly, a lack of anaesthetic monitoring (or partial monitoring without pulseoximetry) was identified as a risk factor for mortality. This, together with oxygen supplementation, which was identified as a protective factor, suggests that hypoxaemia may contribute to the mortalities in some cases. Veterinarians who record a variety of anaesthetic monitoring data may simply identify deteriorating clinical parameters and intervene earlier, thus improving survival rates. Mild to moderate hypoxaemia has been reported in cheetahs anaesthetised with tiletamine-zolazepam together with ketamine and xylazine (Lewandowski, Bonar & Evans 2002) as well as in cheetahs breathing air, while anaesthetised with medetomidine or midazolam in combination with ketamine or tiletamine or zolazepam (Stegmann & Jago 2006). In the latter study, oxygen administration significantly improved the arterial partial pressure of oxygen. When dexmedetomidine was used with midazolam and butorphanol for induction, SpO_2_ decreased over time and more than half of the cheetahs in the study required manual intermittent positive pressure ventilation 30–40 min after induction (Woc Colburn et al. 2017). Hypoventilation and/or hypoxaemia therefore appear to occur in anaesthetised cheetahs in the absence of oxygen supplementation, regardless of the drug combination used for induction. In our survey, 40% of the reported mortalities occurred an hour or more after immobilisation. It is therefore possible that progressive hypoxaemia may have contributed to the death of these individuals. Terminal hyperthermia or hypoxaemia results in few anatomically evident lesions, especially in animals that die acutely. The lesions described in cheetahs that died during or after anaesthesia were suggestive of acute cardiorespiratory failure with possible hypotension or ischaemic tissue damage, often with evidence of dyspnoea. No evidence of pre-existing significant pathology was present in 54% of cases, supporting a conclusion that death was because of acute organ malfunction.

A surprising finding in the survey was the high number of mortalities (30%) that occurred after the anaesthetic antidotes were administered. Stormy recoveries have been reported in cheetahs if the primary α_2_-antagonist atipamezole is given at higher doses than reported in this survey (Woc Colburn et al. 2017) or if it is given intravenously rather than intramuscularly. Recoveries are reported to be smooth and uneventful when either atipamezole or yohimbine is used at lower doses. Significant relapses into sedation after either atipamezole or yohimbine administration have not been reported in the literature. Convulsions associated with ketamine have not been reported to our knowledge in cheetahs subsequent to either atipamezole or yohimbine administration. Cheetahs are frequently transported in crates after the antidotes are administered. The use of the long-acting tranquilliser zuclopenthixol acetate is discouraged because of severe side effects such as inappetence, ataxia and extrapyramidal reactions (Huber, Walzer & Slotta-Bachmayr 2001). In addition, other tranquillisers such as haloperidol and perphenazine are not often used in cheetahs. This was also reflected in our survey in that only a single cheetah that died received a specific tranquilliser (diazepam) prior to transport. The lack of tranquillisation during transport in unhabituated free-ranging cheetahs is likely to result in severe stress. The potential for subsequent hyperthermia is exacerbated by poorly ventilated transport crates. Further research on tranquillisers that can be safely used to reduce anxiety during capture and translocation in cheetahs is therefore of critical importance. In addition to hyperthermia and hypoxaemia, anaesthetic deaths are likely to be multifactorial, with factors such as bronchospasm, laryngeal oedema, pre-existing infectious or non-infectious disease playing a role in some animals. Aspiration of stomach contents was a common terminal finding, occurring in one case when a cheetah was fed a large meal after it recovered.

The study was limited to some extent by the number of questions that we felt we could ask because of concerns that veterinarians would not participate in the survey if it took too long to complete. In retrospect, the addition of a few key questions would have added some important information that would have provided more clarity on certain aspects of the study. For example, we failed to ask veterinarians about the exact number of cheetahs that they had immobilised in their career. If we had that information we would have been able to provide estimated mortality rates. We also failed to ask specific questions about the antidotes (medicines, doses or routes of administration) that were given in cheetahs that died after they were administered. In addition, the 30 deaths reported represent a relatively small number for statistical analysis, especially because almost a third of the reported fatalities were reported by one veterinarian. Nevertheless, the study does provide some important information on factors that can be addressed to reduce capture-related mortalities in this species.

## Conclusion

The survey results suggest that free-ranging rather than habituated captive cheetahs are particularly at risk of dying during immobilisation and transport. The stress associated with capture in these free-ranging animals may make them particularly susceptible to hyperthermia and other anaesthetic complications. The capture-related fatalities in this species do not appear to be associated with either a lack of experience on the part of the attending veterinarian or the particular drug combination used for immobilisation. The survey highlights the need for more active monitoring of cardiovascular and respiratory parameters and the provision of supplementary oxygen, particularly in cheetahs anaesthetised for periods longer than an hour. Anaesthetic monitoring and the management of stress after antidote administration also need to be improved. The availability of a portable isoflurane anaesthetic machine, suitable monitoring equipment, an adequate supply of oxygen and a large supply of cold water and/or ice packs should perhaps be considered essential equipment for veterinarians working with cheetahs in the field.
